# Cerebrospinal fluid NPTXR changes and relationships with β‐amyloid load and metabolism by PET in Alzheimer’s disease

**DOI:** 10.1002/alz70861_108487

**Published:** 2025-12-23

**Authors:** Ming Ni, Xinyi Lyu, Linbin Dai, Feng Gao, Jiong Shi, Yong Shen

**Affiliations:** ^1^ Department of Nuclear Medicine, Division of Life Sciences and Medicine, The First Affiliated Hospital of USTC, University of Science and Technology of China, Hefei, Anhui China; ^2^ Department of Neurology, The First Affiliated Hospital of USTC, Division of Life Sciences and Medicine, University of Science and Technology of China, Hefei, Anhui China; ^3^ Department of Neurology, Institute on Aging and Brain Disorders, The First Affiliated Hospital of USTC, Division of Life Sciences and Medicine, University of Science and Technology of China, Hefei, Anhui China; ^4^ Neurodegenerative Disorder Research Center, Division of Life Sciences and Medicine, University of Science and Technology of China, Hefei, Anhui China

## Abstract

**Background:**

Neuronal pentraxin receptor (NPTXR) has been identified as a cerebrospinal fluid (CSF) biomarker associated with synaptic pathology, demonstrating its significant correlation with progressive cognitive impairment in Alzheimer’s disease (AD). This study investigated the changes of CSF NPTXR in AD patients, while explored its associations with cerebral amyloid‐β (Aβ) deposition and glucose metabolism by positron emission tomography (PET) imaging.

**Method:**

TThis retrospective investigation enrolled 59 AD cases from the China Aging and Neurodegenerative Disorder Initiative (CANDI) cohort, with age‐matched cognitive normal (CN, n = 17) and non‐AD dementia (non‐ADD, n = 20) controls. AD group was further stratified into three subgroups using clinical dementia rating (CDR) score: mild cognitive impairment (MCI, CDR = 0.5, n = 17), mild AD dementia (CDR = 1, n = 29), and moderate to severe AD dementia (CDR = 2‐3, n = 13). In the AD group, all patients underwent ^18^F‐florbetapir and ^18^F‐FDG PET to evaluate cerebral Aβ deposition and glucose metabolism.

**Result:**

CSF NPTXR concentrations in both AD groups and non‐ADD group were significantly lower than that in the CN group (p < 0.001). CSF NPTXR was significantly different between MCI and mild AD (p < 0.016), as well as mild and moderate‐severe AD (p < 0.001). In the AD group, CSF NPTXR demonstrated enhanced correlations with core AD CSF biomarkers, showing significant positive associations with *p* ‐tau181 (r = 0.442, *p* < 0.001), T‐tau (r = 0.420, *p* < 0.001), and Aβ40 (r = 0.463, *p* < 0.001), while exhibiting a negative correlation with the Aβ42/Aβ40 ratio (r = ‐0.545, *p* < 0.001). Voxel‐based analysis revealed that CSF NPTXR showed positive correlations with ^18^F‐florbetapir PET in the bilateral medial temporal lobe (including the hippocampus and parahippocampal cortex), and with ^18^F‐FDG PET in the bilateral lateral temporal lobe, parietal lobe (predominantly the precuneus and posterior cingulate cortex), and frontal lobe.

**Conclusion:**

Decreased CSF NPTXR concentration exhibits strong associations with the severity of AD. NPTXR may play an important role in mediating Aβ pathology driven synaptic dysfunction and subsequent neuronal loss in AD.

